# Fe-DCA Metal–Organic Frameworks on the Bi_2_Se_3_(0001) Topological Insulator Surface

**DOI:** 10.1021/acsomega.6c01621

**Published:** 2026-05-14

**Authors:** Anna Kurowská, Jakub Planer, Pavel Procházka, Veronika Stará, Elena Vaníčková, Zdeněk Endstrasser, Matthias Blatnik, Čestmír Drašar, Jan Čechal

**Affiliations:** † CEITEC–Central European Institute of Technology, 48274 Brno University of Technology, Purkyňova 123, Brno 612 00, Czech Republic; ‡ 48252 University of Pardubice, Studentská 95, Pardubice 53210, Czech Republic; § Institute of Physical Engineering, Brno University of Technology, Technická 2896/2, Brno 616 69, Czech Republic

## Abstract

The formation of two-dimensional metal–organic frameworks (MOFs) on the surface of a topological insulator (TI) is a pathway to engineer quantum materials with exotic properties. MOFs featuring ferromagnetically coupled metal atoms are theoretically predicted to induce an exchange gap in the topological surface states, potentially leading to a quantum anomalous Hall effect. However, achieving ordered MOFs on TI surfaces remains challenging because of the limited knowledge of self-assembly on these substrates. In this paper, we demonstrate the self-assembly of Fe atoms and dicyanoanthracene (DCA) molecules into 2D MOFs on the Bi_2_Se_3_(0001) surface at room temperature, investigated via a combination of low-energy electron microscopy and diffraction (LEEM/LEED), scanning tunneling microscopy (STM), and ab initio calculations based on density functional theory (DFT). Two competing Fe-DCA phases formed. The first phase corresponds to a close-packed Fe_1_DCA_3_ structure. In contrast, the second phase exhibits a larger unit cell with no match to either known or DFT-calculated systems, indicating a more complex bonding environment. These findings advance the understanding of the growth of MOFs on a strong topological insulator surface and provide insights into designing MOF/TI interfaces with tailored electronic and magnetic properties.

## Graphical abstract

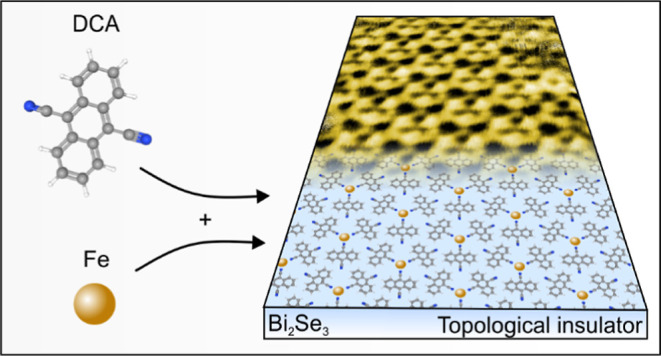

## Introduction

Topological insulators (TIs) belong to a class of materials having an insulating bulk and conductive surface states protected by time-reversal symmetry (TRS).^1,2^ TIs were originally proposed in time-reversal-invariant systems; nonetheless, the onset of spontaneous magnetization, which breaks TRS, is expected to enable unconventional phenomena associated with a nontrivial topology.^3–7^ Spontaneous magnetization of TIs can emerge due to the proximity of a ferromagnetic (FM) order, such as a layer of magnetically coupled metal atoms, and open an exchange gap in the Dirac band dispersion while preserving the bulk topology and energy landscape. This could enable dissipationless charge transport, spin currents, and the emergence of Majorana Fermions, with potential applications ranging from spintronics to topological quantum computation.^3,4,6^

Placing a properly designed magnetically coupled 2D layer on the TI surface thus presents a viable strategy to control the quantum states of matter and achieve new properties. In this context, on-surface coordination chemistry offers a powerful approach.^8,9^ In 2D metal–organic frameworks (MOFs), organic linkers ensure the periodic order of transition metal (TM) atoms within a single layer on the surface, which enables their functionality as single-atoms catalysts,^10,11^ host ferromagnetism,^12^ or topologically nontrivial states.^13–16^ The local magnetic moments of TM atoms embedded in MOFs are theoretically predicted to remain unquenched, enabling the necessary exchange interaction to break TRS spontaneously.^17^ Combined with flexible design, widely tunable properties, and an ability to form large-scale structures via self-assembly, MOFs are an ideal candidate to explore the interplay of strictly 2D magnetism and topological surface states, providing perspectives for realizing a robust quantum-anomalous-Hall and Majorana effects.^6^

The self-assembly of organic and metal–organic structures is well-studied on metal surfaces,^8,9^ yet transferring this knowledge of molecular systems from metals to vdW material surfaces is often far from being straightforward, as subtle parameter changes can strongly influence the resulting structure and targeted functional properties. For nonmetallic substrates, most progress has been made on epitaxial graphene,^11,18–22^ followed by hBN^18,23^ and NbSe_2_ ^24,25^ substrates. However, preparing a single-layer MOF on the TI surface remains a significant challenge. Here we employ a combination of low-energy electron microscopy and diffraction (LEEM/LEED), scanning tunneling microscopy (STM), and ab initio calculations based on density functional theory (DFT) to explore the formation of 2D MOFs from Fe atoms and 9,10-dicyanoanthracene (DCA) molecules (Figure 1).

**Figure 1:**
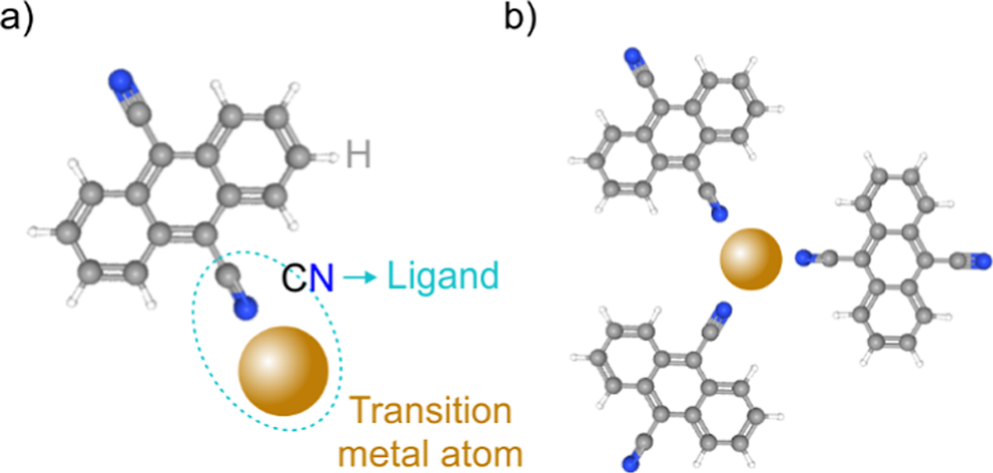
(a) Structure of a DCA molecule forming a coordination bond with a TM atom and (b) the resulting clover-leaf motif.

Among molecular and metal–organic systems studied on topological insulator substrates, metal phthalocyanines and porphyrins^26–39^ have received the most attention, while other systems like TCNQ and 4F-TCNQ,^40,41^ TTF,^42^ alkanes,^43^ fullerenes,^44^ PTCDA,^45^ and BDA^46^ have been explored to a lesser extent. Notably, TCNQ,^40^ PTCDA,^45^ and BDA^46^ form a highly ordered two-dimensional molecular network that weakly interacts with the substrate. In contrast, the deposition of 4F-TCNQ molecules resulted in a downward shift of the Fermi level, indicating significant charge transfer. Concerning the metal phthalocyanines (Pc), the central metal atom determines the interaction strength with the substrate and, consequently, the alignment of Pc molecules. On Bi_2_Te_3_, a clear trend is observed: MnPc shows strong surface interaction accompanied by the formation of an interfacial dipole, which suppresses surface molecular mobility and hinders the formation of ordered superstructures.^29,33,38^ In contrast, CoPc, CuPc, and FePc form well-ordered molecular superstructures, with CoPc adopting a quasi-hexagonal lattice and CuPc^29^ and FePc^27^ forming square lattices with two distinct orientations, whose appearance is modulated by the extended unit cell. This trend indicates a weakening of the substrate interaction from Mn to Co, Cu, and Fe. Changing the substrate to Bi_2_Se_3_ leads to remarkable differences: while CoPc^26^ still forms a quasi-hexagonal arrangement, MnPc^36^ now assembles in an ordered fashion, and FePc^35^ and CuPc^37^ do not show any ordering. The stronger interaction of metal atoms with Bi_2_Se_3_ was attributed to the larger electron affinity of Se, which favors charge transfer from the molecules to the substrate and reduces the metal-surface distance.^26^ This also signifies the role of the substrate, as even closely related Bi_2_Se_3_ and Bi_2_Te_3_ substrates can dramatically alter the adsorption properties.

Considering 2D MOFs, some progress has been made with Co-DCA on the Bi_2_Te_3_ surface, where small patches of the mixed honeycomb-kagomé (MHK) lattice form but are unstable above −20 °C.^47^ Apart from that, however, no other MOF formation has been reported on TI substrates. While molecular ligands weakly interact with TI surfaces,^46^ the metal atoms can interact strongly with the substrate atoms. This brings complexity to the system as metal atoms could have preferential adsorption sites or can form competing compounds like FeSe.^48^ On top of these kinetic and thermodynamic aspects of the growth, the interaction with the substrate can change the functionality of the prepared structure. While some interaction is crucial to disturb the TI surface states, a too strong interaction with the substrate can result in the loss of intrinsic properties of the MOF. In this study, the use of DCA molecules and Fe atoms as suitable building blocks (see Figure 1 for the binding motif) for the formation of MOFs on the Bi_2_Se_3_ surface is investigated. DCA has been widely reported to form MOFs with various TM atoms on different surfaces;^12,24,25,49–56^ some of these are predicted to exhibit magnetic properties. Moreover, recently, Lobo-Checa et al. demonstrated that Fe_2_DCA_3_ with an MHK lattice on Au(111) displays ferromagnetic coupling up to 35 K,^12^ making the Fe-DCA system a promising candidate for such studies.

In this paper, we investigated the formation of Fe-DCA 2D MOFs on the surface of a Bi_2_Se_3_ topological insulator substrate at room temperature. Using a combination of LEEM, LEED, and STM, we observed two distinct Fe-DCA phases. The unit cell parameters, determined from the experimental data, were compared to those reported in the literature and calculated by DFT. While one phase matches the parameters of a close-packed structure, the second does not correspond to any previously reported or DFT-calculated structures, suggesting a more complex bonding environment. Our analysis indicates that Fe-DCA does not assemble into the MHK lattice under our experimental conditions but instead forms an alternative structure influenced by substrate templating effects and by the growth kinetics.

## Results and Discussion

Deposition of DCA molecules (Figure 1a) and Fe atoms onto a clean Bi_2_Se_3_ surface at room temperature results in the formation of a metal–organic structure. Details on the characterization of the as-prepared substrate, including confirmation of its Se termination by low-energy ion scattering spectroscopy and LEEM-IV, are provided in the Supporting Information, Sections 1 and 2. The LEEM bright-field image (Figure S3 in Supporting Information) shows a reduced contrast, suggesting the presence of an overlayer after the deposition, while the diffraction pattern in Figure 2a clearly shows additional spots attributed to the newly formed superstructure. The sharpness of the diffraction patterns shown in Figure 2a,c,d indicates long-range order and reflects the quality of the grown structure. Analysis of the diffraction patterns described below reveals two distinct phases on the surface, denoted A and B. Phase B is relatively easy to obtain at a low DCA deposition rate (lower part of the interval 0.02–0.2 ML/min), and it is the only phase present for both submonolayer and full monolayer coverages. In contrast, phase A requires a high deposition rate of DCA and Fe, and it was observed only as a phase coexisting with phase B at or above full monolayer coverage. Upon gradual heating to 80 °C, the diffraction patterns of both phases disappear, indicating a loss of long-range order, likely due to decomposition of the metal–organic phases and desorption of DCA molecules. On a sample with coexisting phases A and B, the spots corresponding to phase A disappear at a comparatively lower temperature than those of phase B, indicating that phase B is more stable.

**Figure 2:**
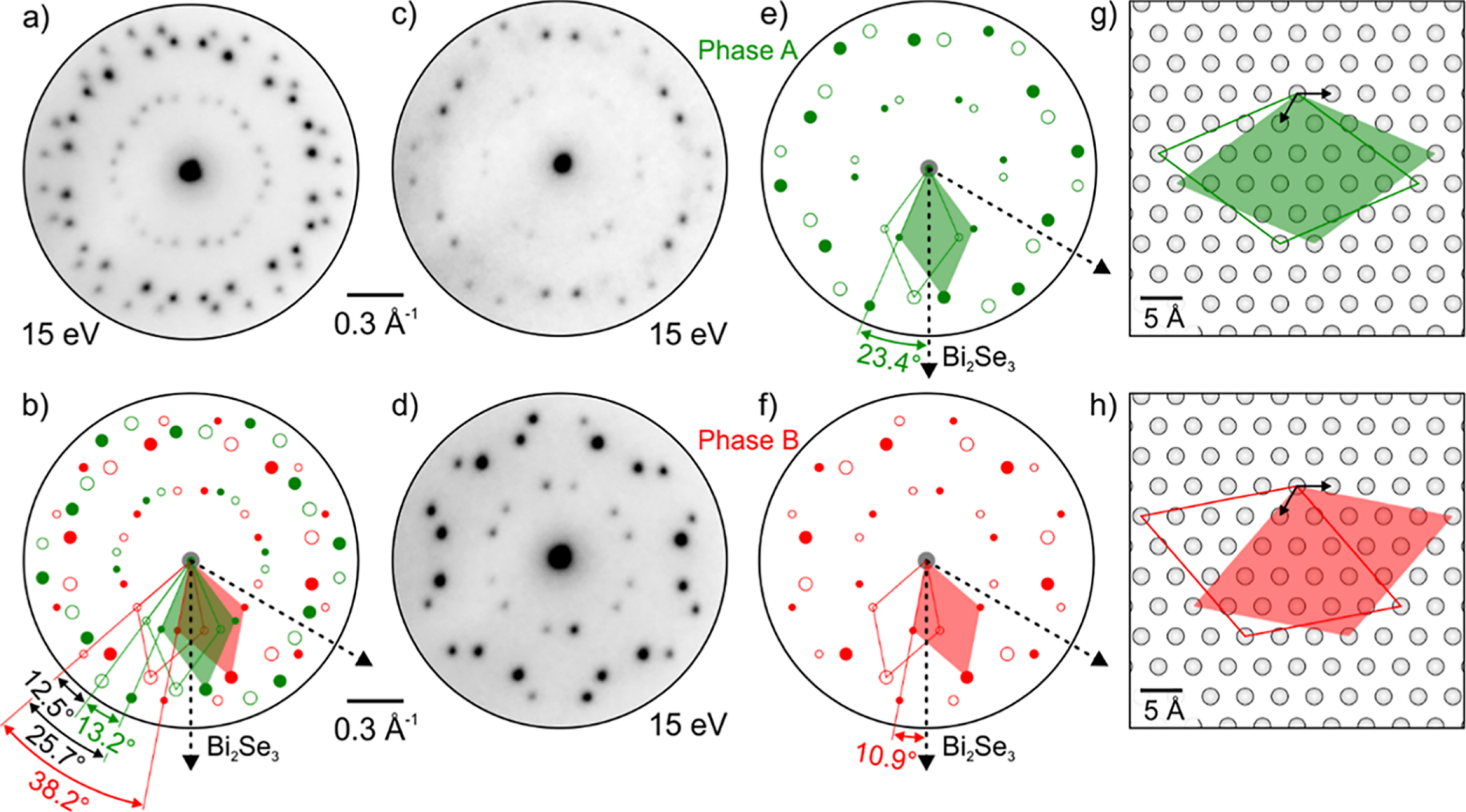
Measured and modeled diffraction patterns of Fe-DCA on the Bi_2_Se_3_(0001) surface. (a) Diffraction pattern of both phases A and B present on the surface. (b) Model of the diffraction pattern in (a). Colored arrows and parallelograms represent unit cells of phases A (green) and B (red); the gray arrows denote principal substrate directions of Bi_2_Se_3_. (c,d) Microdiffraction patterns measured on a circular area with a diameter of 3.7 μm showing the majority of phase A (c) and only phase B (d). (e,f) Diffraction models corresponding to pure phases A and B with highlighted rotation with respect to the substrate. (g,h) Real space model obtained by the Fourier transform of diffraction models.

The diffraction models of phases A and B shown in Figure 2b,e,f were obtained by modeling the diffraction pattern in ProLEED Studio^57^ using the measured microdiffraction patterns showing a sample area with pure phase B (Figure 2d) and the majority of phase A (Figure 2c). Lattices of phases A and B are commensurate with the substrate and are described in matrix notation as $\left(\begin{array}{cc} 3 & -2\\ 2 & 5 \end{array}\right)$ and $\left(\begin{array}{cc} 4 & -1\\ 1 & 5 \end{array}\right)$ , respectively. The associated real-space unit cells are depicted in Figure 2g and h, respectively. The lengths of the real-space unit cell vectors (referred to as unit cell size in the following) of phases A and B are 18.1 Å and 19.0 Å, respectively. Thus, the unit cell size of phase B is 5% larger than that of phase A. These phases have different orientations with respect to the principal Bi_2_Se_3_ substrate directions: phase A is rotated by 23.4° and phase B by 10.9° (see Figure 2e,f). In summary, phase B has a larger unit cell is more stable than phase A. Because phase A is observed only within a layer of Fe-DCA covering the entire surface, we infer that it is a compressed phase.

Next, we present the STM results for the samples previously analyzed by LEEM in Figure 2, showing both structures coexisting on the surface. The STM image in Figure 3a shows an array of trigonal protrusions (see details in Figure 3b); due to their visual appearance, we refer to this motif as a clover-leaf motif. We assign the clover-leaf motif to an Fe atom coordinated with three DCA molecules, as schematically depicted in Figure 1b. These clover-leaf motifs are arranged in a hexagonal lattice, with all the motifs appearing symmetrically and oriented in the same way within a single domain. We note that the STM images were challenging to acquire due to the weak interaction of DCA with the Bi_2_Se_3_ substrate.^46^ Room-temperature measurements were possible only on the sample with full monolayer coverage (presented in Figure 3); for submonolayer coverages, the samples had to be cooled to −100 °C to achieve atomic/molecular resolution (see Figure S4 in Supporting Information). The clover-leaf motif was the most prevalent, although at specific sample biases or under different tip conditions, other appearances were also observed, as shown in Figure S5 in the Supporting Information and in the literature.^58^

**Figure 3:**
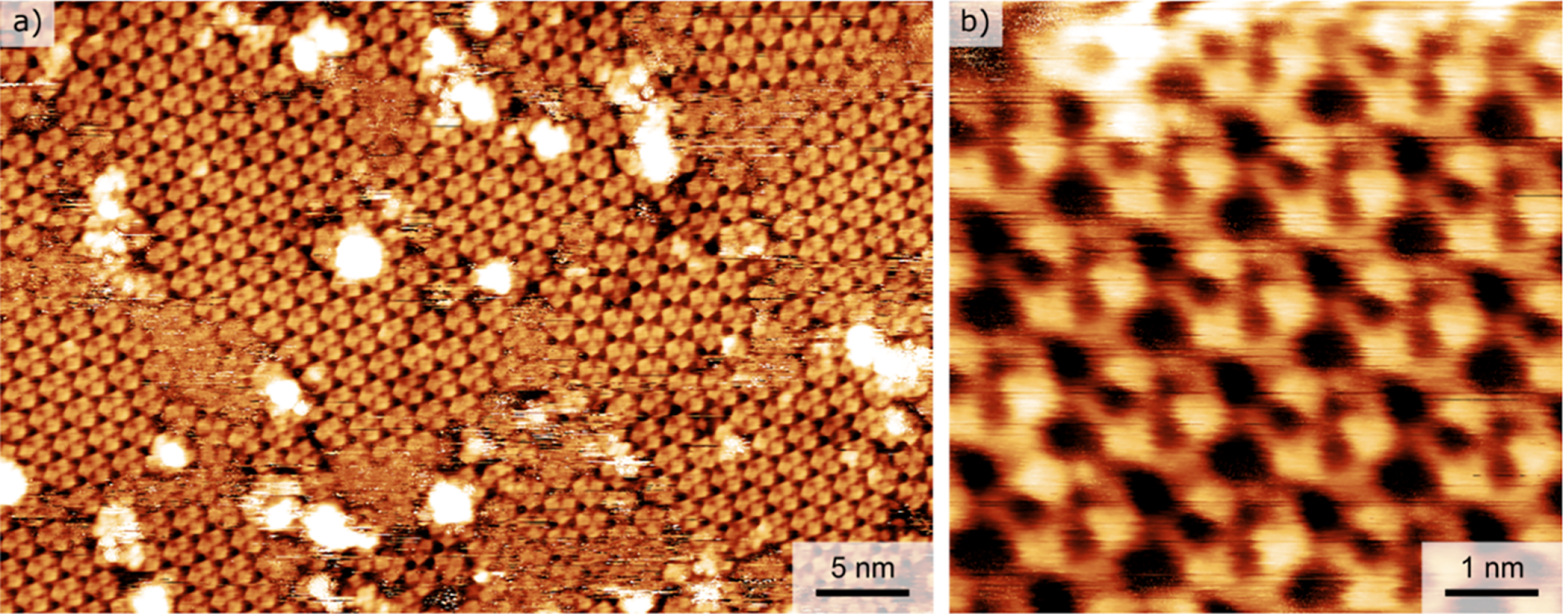
High-resolution STM images of the Fe-DCA structure on the Bi_2_Se_3_(0001) surface. (a) Large-scale image showing two domains of clover-leaf motifs arranged in a hexagonal lattice; the overly bright protrusions are probably clusters of Fe atoms. (b) Detailed view of the lattice of clover-leaf motifs. Scanning parameters: (a) 1.0 V, 30 pA and (b) 1.8 V, 30 pA.

To associate the clover-leaf motif with lattices A and B, we have measured angles between the orientations of the clover-leaf motifs and their assembly in distinct rotational/mirror domains, as demonstrated in Figure 4. Two distinct sets of angular differences were observed: approximately 38° corresponding to two domains of phase B (38.2°), see Figures 2b and 4a, and approximately 10° corresponding either to two domains of phase A (13.2°) or to an interphase of A and B (12.5°), see Figures 2b and 4b. For the latter, a 5% difference in unit-cell size between the two phases is within the resolution uncertainty/error of the room-temperature STM, thus it will not allow us to distinguish the phases precisely. The results presented above suggest that both phases A and B have similar clover-leaf motifs, featuring 3-fold-coordinated Fe with three DCA molecules.

**Figure 4:**
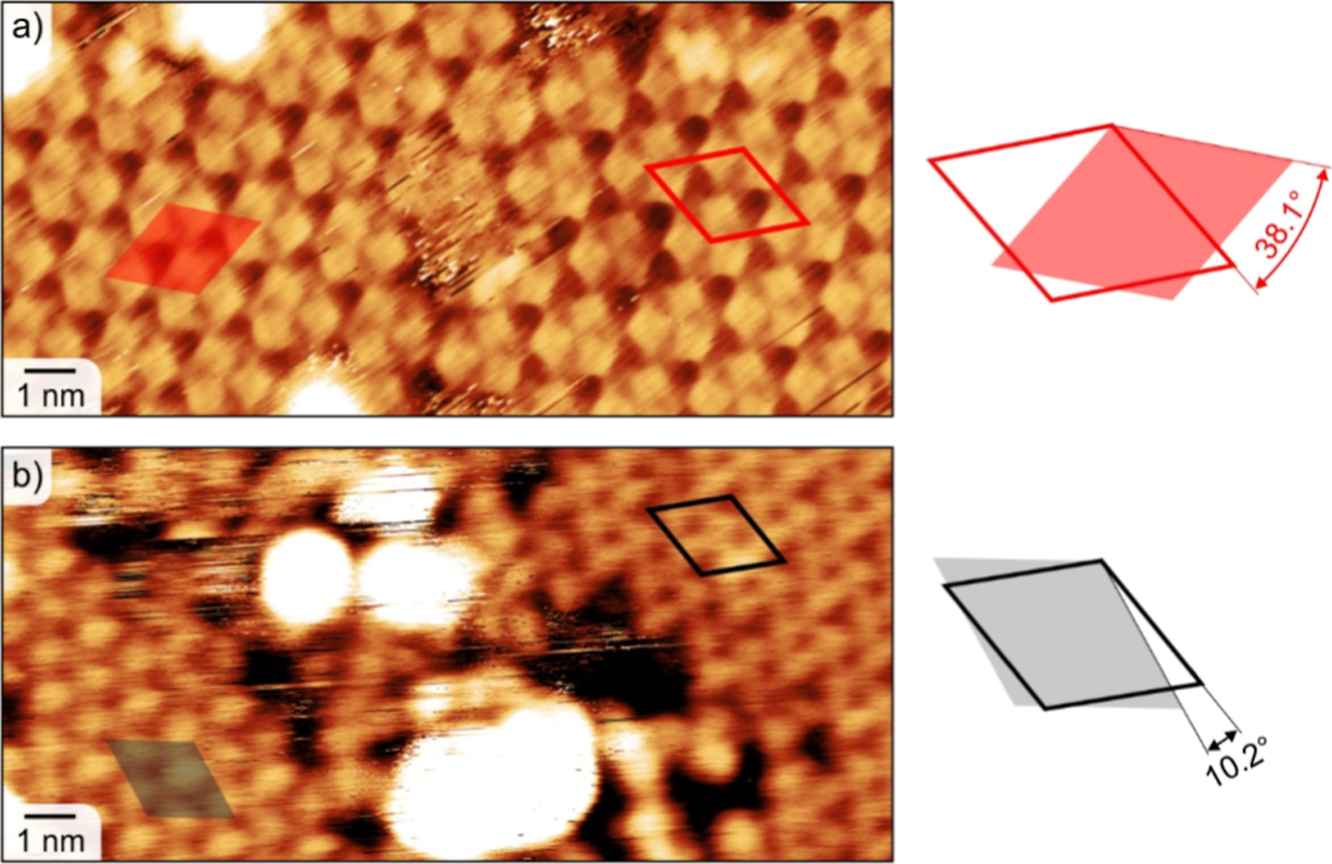
Angle analysis of Fe-DCA on Bi_2_Se_3_(0001) in STM images. (a) STM image showing two rotational domains of phase B. (b) STM image showing either two rotational domains of phase A, or the interphase of A and B. Reference for angles between two symmetry-equivalent domains is provided at the side of the STM images. Scanning parameters: (a) 1 V, 30 pA and (b) 1.8 V, 30 pA.

To confirm the structure and composition of phases A and B, we have performed DFT calculations. There, the substrate is modeled as a single quintuple layer of Bi_2_Se_3_, and spin–orbit coupling is excluded for its high computational cost.^46^ As initial models for the calculations, we have considered the clover-leaf motif for Fe_1_DCA_3_ forming a close-packed lattice, assuming the experimental lattices shown in Figure 5a,b. The optimized structure of Fe_1_DCA_3_ was calculated to have a unit-cell vector of 17.95 Å, which closely matches the experimental value of 18.1 Å and the phase-A orientation. Within this structure, the Fe_1_DCA_3_ motifs are bound together through hydrogen bonds (see Figure 5c). In contrast, phase B is larger with an experimental unit cell size of 19.0 Å, as given in Figure 5b. The total energy of the calculated gas-phase model, which matches the experimental size of the phase A, is 300 meV per Fe_1_DCA_3_ formula unit higher than the gas-phase model expanded to match the unit cell of the phase B, which is caused primarily due to the weakening of hydrogen bonds between the Fe_1_DCA_3_ units. Furthermore, the adsorption energy of the close-packed phase on the Bi_2_Se_3_ substrate is 120 meV more favorable in the unit cell of phase A than in that of phase B, which results in a 14% lower adsorption energy per unit area for the close-packed phase in the unit cell of phase A. The DFT calculations thus suggest that phase A should be the preferred close-packed phase, whereas the close-packed structure is not stable in the unit cell of phase B without an additional stabilization mechanism, which contradicts the ease of preparation of phase B. In the unit cell of phase B, there are two additional points equivalent to the position of the corner metal atoms, as highlighted in Figure 5b, which can potentially host an additional component. However, despite many efforts, such as including Fe, Bi, or Se adatoms in computations, we did not obtain any stable structure. Computational inclusion of Fe and Bi yielded the twisted MHK structure described below, whereas inclusion of Se showed low binding strength and led to a low-stability structure, see Supporting Information, Section 7.

**Figure 5:**
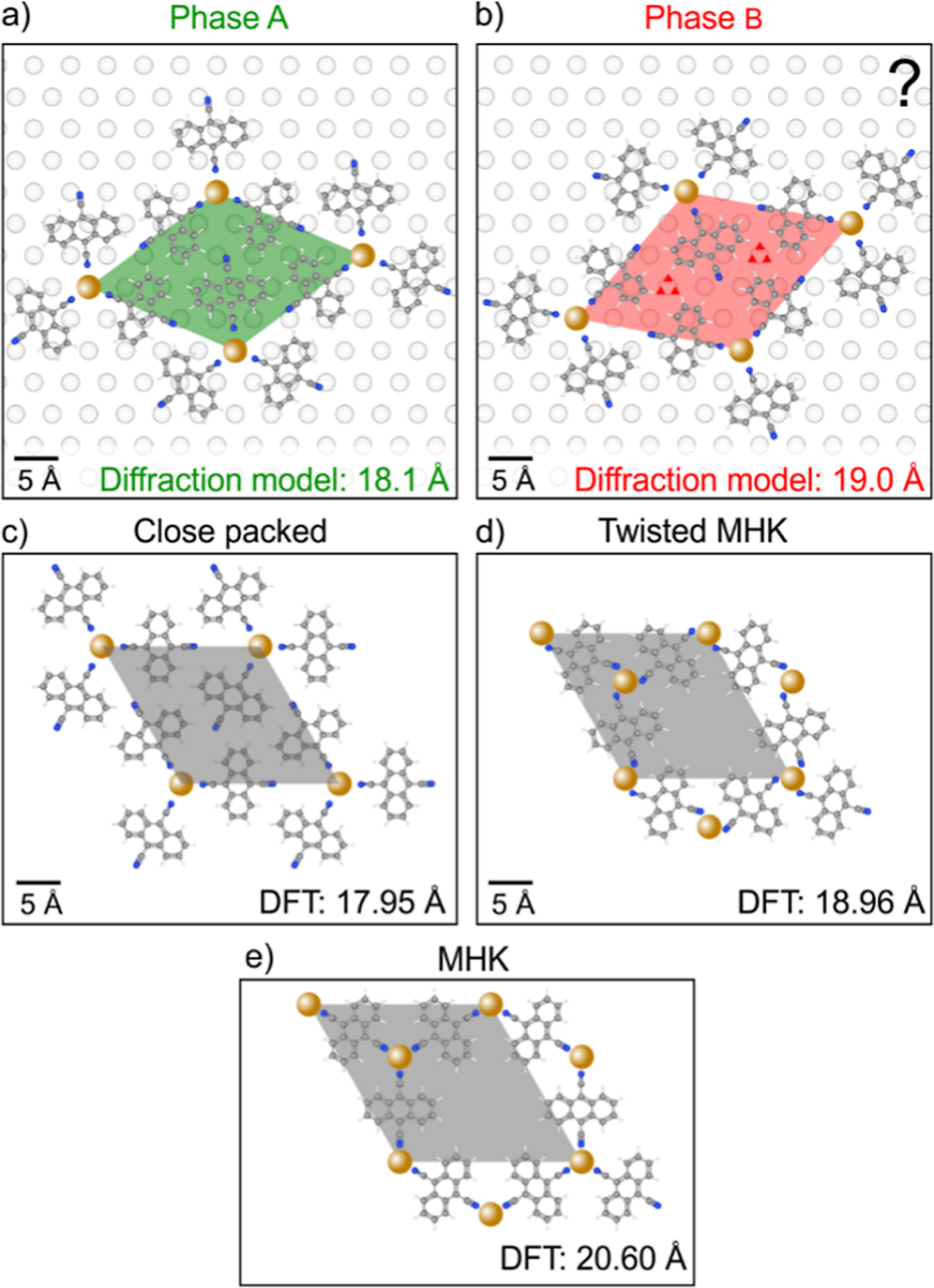
(a, b) Experimental unit cells of phases A and B determined by modeling of the diffraction patterns; the corners of the unit cells were arbitrarily positioned on the substrate lattice points. (c) Gas phase DFT model of Fe_1_DCA_3_ structure showing hydrogen-bonded clover-leaf motifs. (d) Calculated gas phase structure of Fe_2_DCA_3_ on Bi_2_Se_3_ showing twisted DCA molecules within the MHK lattice. (e) Gas phase DFT model of Fe_2_DCA_3_ with an MHK lattice.

The calculated free-standing Fe_2_DCA_3_ with a MHK has a unit cell size of 20.6 Å, see Figure 5e. DFT optimization of Fe_2_DCA_3_ placed on Bi_2_Se_3_ results in a structure where the DCA molecules are twisted around the central atoms, suggesting that the nontwisted MHK is not energetically favorable/stable on Bi_2_Se_3_. The twist causes shrinkage, allowing the equivalent positioning of all Fe atoms relative to the substrate atoms beneath. Such a structure would have a unit cell size of 18.96 Å, as shown in Figure 5d. DFT calculations thus show that the 2D MOF is defined by the position of the metal atom with respect to the substrate adsorption site (a position above the Se atom is preferred), which is in line with the behavior of metal phthalocyanines on Bi_2_Se_3_ and Bi_2_Te_3_ described in the introduction.

The measured real-space unit cell size of phase A is 18.1 Å, which fits well with the calculated Fe_1_DCA_3_ motif. We considered the hypothesis that phase B is Fe_2_DCA_3_ with the MHK lattice, but our experimental observations do not support this. Although the measured real-space unit cell size of phase B (19.0 Å) matches the calculated twisted MHK unit cell (18.96 Å), all STM images show phase B to be highly symmetric, which contradicts the twisted motif found in calculations. Furthermore, the MHK lattice is expected to have not only a larger unit cell size but also an interwoven structure (as seen in our results from Au(111) in Figure S6 in the Supporting Information), which is absent in the majority of our STM images, where phase B displays a clover-leaf motif. Even though MHK is sometimes imaged similarly to a clover-leaf motif, both have very distinct orientations of the motifs within the lattice, as detailed in the Supporting Information, Section 6. Therefore, we reject the hypothesis that phase B is MHK. The experimental results suggest that phase B also has Fe_1_DCA_3_ close-packed structure, but with a larger unit cell than phase A. However, we could not find a stabilization mechanism that would computationally support its existence.

In the literature, there are two distinct motifs reported for metal (M) and DCA (M_2_DCA_3_ and M_1_DCA_3_) at a variety of substrates, i.e., Cu(111),^49–51^ Ag(111),^52^ Au(111),^12,53^ Gr/Ir(111),^54,55^ hBN,^56^ NbSe_2_.^24,25^ The M_2_DCA_3_ has a fully reticulated structure with an MHK lattice, which typically forms an 8 × 8 superstructure with respect to the substrate; reported unit cell sizes range from 19.6 to 21.1 Å. The only exception is provided by Pawin et al., who reported Cu_2_DCA_3_ on Cu(111) with a unit cell size of 17.9 Å and a 7 × 7 superstructure,^50^ which is likely caused by strong interactions between the molecules and the substrate. If we compare the measured phase B size of 19.0 Å with reported MHK lattices, we conclude that it is significantly smaller than expected for MHK, further supporting our conclusion that phase B is not MHK. The second motif, a close-packed lattice of hydrogen-bonded M_1_DCA_3_ clover-leaf units, is assumed to form at higher coverages as a consequence of filling the porous M_2_DCA_3_ framework with excess DCA and subsequent transformation. It is not as prevalent in literature as MHK. Cu_1_DCA_3_ on Cu(111) is reported to have a $4\sqrt{3}\times 4\sqrt{3}\;R30^{{}^{\circ}}$ superstructure, with a unit cell size of 17.7 Å.^50,58^ This number is very close to our experimental (phase A) and DFT calculated (close-packed on Bi_2_Se_3_) unit cell sizes. Conversely, Ni_1_DCA_3_ on NbSe_2_ shows a close-packed-like structure where the whole clover-leaf motifs are twisted by a small angle within the unit cell with a size of 20.03 Å.^25^ Since this is an even larger unit cell size than the phase B, the hydrogen bonding should also be significantly weakened here; however, the additional stabilization mechanism was not reported.

The Bi_2_Se_3_ substrate was recently reported to exhibit very low interaction strength with adsorbed organic molecules.^46^ In contrast to graphene, which can induce the epitaxial ordering through π–π interactions, the templating effect on Bi_2_Se_3_ is much weaker. As a result, molecular layers on Bi_2_Se_3_ must be stabilized primarily by intermolecular interactions strong enough to prevent molecule detachment and desorption. However, for 2D MOFs, metal atoms may interact strongly with the Bi_2_Se_3_ substrate and occupy well-defined adsorption positions, as highlighted for metal phthalocyanines and porphyrins in the introduction. For instance, a strong affinity of Fe atoms toward Se was reported, forming FeSe complexes.^48^ The strong preference for specific adsorption sites may require the metal–organic structure to be in an epitaxial relationship with the substrate to ensure that metal atoms occupy their favorable positions. Therefore, primarily 2D MOFs in which all metal atoms are in their favorable adsorption sites can be expected to be stable on the Bi_2_Se_3_(0001) substrate.

## Conclusion

In conclusion, we reported the synthesis of 2D metal–organic frameworks comprising Fe atoms and DCA molecules on the surface of a topological insulator, Bi_2_Se_3_(0001). DCA coordinates with Fe atoms and forms two distinct phases, A and B, which differ in the unit cell size. We have assigned phase A to be a close-packed structure, reported for metal-DCA MOFs on various substrates. In the case of phase B, neither reported nor DFT-calculated structures show a match, pointing to a more complex bonding environment. Our analysis indicates that Fe-DCA does not assemble into the MHK lattice under our experimental conditions but instead forms an alternative structure influenced by kinetic and substrate effects and atom positions. Nonetheless, it remains possible that, despite a missing MHK symmetry, the close-packed structure can still exhibit magnetic coupling as was observed on NbSe_2_.^25^ Our findings nevertheless provide insights into the formation of metal–organic structures on topological insulator surfaces, paving the way toward the synthesis of hybrid organic–inorganic material systems with the prospect of forming magnetic topological insulators via on-surface synthesis.

## Methods

All measurements were carried out at the UHV cluster of the CEITEC Nano Core Facility. The cluster consists of a UHV transfer line and several different end stations. This allows samples to be cleaned, prepared, and characterized using multiple complementary techniques without exposure to ambient conditions. The base pressure in the UHV transfer line is 2 × 10^–10^ mbar; during sample transfers (60–180 s), the pressure increases to 2 × 10^–9^ mbar but quickly recovers after the movement has ceased. All chambers are pumped by standard UHV pumping equipment (turbomolecular, ion-getter, and Ti-sublimation pumps).

## Sample Preparation

The **Bi**_2_ **Se**_3_ **samples** were fabricated by heating stoichiometric 5N mixtures of Bi and Se (both from Sigma-Aldrich/Merck) with the FMC method^46^ at the University of Pardubice, CZ. The resulting crystals are 4–8 mm in length, 3–6 mm in width, and up to 3 mm thick, and were mounted on a specially designed sample holder, allowing in situ exfoliation of the crystals within the UHV cluster. The process of in situ exfoliation is described in detail in our previous paper.^46^ **DCA molecules are deposited** by a Createc Near-Ambient Effusion Cell from a quartz crucible at a process temperature of 55–75 °C on the sample(s) held at room temperature. The molecular powder was purchased from Sigma-Aldrich/Merck and deposited after thorough degassing under UHV. The deposition rate was not reproducible with temperature over a longer time scale; thus, it was calibrated by depositing on an Ag(100) substrate prior to each deposition on Bi_2_Se_3_. Deposition rates ranging from 0.02 to 0.2 ML/min were tested and used. **Fe atoms are deposited** by a high-temperature cell (MBE Komponenten, WEZ) from a resistively heated quartz crucible at temperatures of 1030 °C on the sample(s) held at room temperature. The Fe pellets were purchased from Mateck and Fe was deposited after thorough UHV degassing. The deposition rate was calibrated using a quartz crystal microbalance to 0.01 pm/s; this corresponds approximately to 0.002 ML/min, while rough calculations indicate that 0.02 ML of Fe is needed for full layer coverage of the Fe_2_DCA_3_ MOF. In the MOF formation, the precise timing and balance of the deposition rates of both components is crucial. Due to the weak interaction between DCA and Bi_2_Se_3_ and, consequently, the low sticking coefficient, the Fe-DCA growth is performed by simultaneous deposition, usually for 10–20 min, with possible additional predeposition of DCA and possible additional postdeposition of DCA or Fe.

### Sample Analysis

**STM** was performed on a commercial Aarhus 150 system (SPECS) with a mounted Kolibri Sensor or a basic tungsten tip in constant-current mode at room temperature (base pressure of 2 × 10^–10^ mbar). The corresponding STM imaging parameters are provided in the respective figure captions. **Low-energy electron microscopy/diffraction (LEEM/LEED)** images were obtained using the SPECS FE-LEEM P90 system (base pressure of 2 × 10^–10^ mbar). Diffraction patterns are formed by collecting signals from a surface area of 15 × 10 μm^2^. For microdiffraction analysis, this area is restricted by a mechanical aperture to a spot size of 3.7 μm. The bright-field images are obtained by the electrons from the (0,0) diffracted beam.

### Theory

Spin-polarized DFT calculations were performed with the Vienna ab initio Simulation Package (VASP)^60^ using the projector augmented wave method (PAW)^61^ to treat core electrons. We used the PBE functional^62^ and Grimme’s pairwise D3 dispersion corrections^63^ to describe the exchange–correlation energy, and a Hubbard-like Coulomb repulsion correction *U*–*J* = 4 eV in Dudarev’s formulation^64^ was considered for an appropriate description of Fe 3d orbitals. The energy cutoff for the plane-wave basis set was set to 500 eV. The Brillouin zone was sampled with a single Γ point. Structural optimizations were stopped when all residual forces acting on atoms in a system were smaller than 0.01 eV/Å. Following our previous work,^46^ the effect of spin–orbit coupling was neglected. All interface models of Bi_2_Se_3_(0001) are composed of a single quintuple layer (QL), which showed a negligible difference in calculated surface energy (<3 × 10^–3^ meV/Å^2^) with respect to the two-QL-thick slab. Additionally, a 15 Å thick vacuum layer was added in the direction perpendicular to the substrate to avoid interactions between periodically repeated replicas. All calculations account for dipole corrections to both energy and forces.

## Supplementary Material



## Data Availability

The data that support the findings of this study, including LEEM/LEED, STM, LEIS, and DFT models, are available at Zenodo at 10.5281/zenodo.19225148.

## Notes

LEEM analysis of UHV-exfoliated Bi_2_Se_3_(0001); determination of Bi_2_Se_3_ surface termination; bright-field image of Fe-DCA on Bi_2_Se_3_(0001); STM for submonolayer Fe-DCA coverages; STM images of Fe-DCA with different tip conditions; mixed honeycomb-kagomé Fe_2_DCA_3_ on Au(111); and off-stoichiometric candidate structures for the phase B (PDF)

## ∥

Department Applied Mathematics and Physics, FH Technikum Wien, Höchstädtplatz 6, 1200 Wien, Austria
