# Supplementary material for: Fe-DCA Metal–Organic Frameworks on the Bi2Se3(0001) Topological Insulator Surface
Source: ACS Omega. 2026 May 14;11(20):30036–44. doi: 10.1021/acsomega.6c01621 (PMC13216953; doi:10.1021/acsomega.6c01621)
Supplement: Supplementary file 1 [file ao6c01621_si_001.pdf]

SUPPORTING INFORMATION

Fe-DCA Metal–Organic Frameworks on the Bi_2Se_3(0001) Topological Insulator Surface

Anna Kurowská,[1] Jakub Planer,[1] Pavel Procházka,[1] Veronika Stará,[1] Elena Vaníčková, [1] Zdeněk Endstrasser, [1] Matthias Blatnik,[1†] Čestmír Drašar,[2] Jan Čechal[1,3]*

[1] CEITEC - Central European Institute of Technology, Brno University of Technology, Purkyňova 123, 612 00 Brno, Czech Republic.

[2] University of Pardubice, Studentská 95, 53210 Pardubice, Czech Republic.

[3] Institute of Physical Engineering, Brno University of Technology, Technická 2896/2, 616 69 Brno, Czech Republic.

AUTHOR INFORMATION

Corresponding Author

* E-mail: cechal@vutbr.cz (J. Č.)

[†] Present Addresses: Department Applied Mathematics and Physics, FH Technikum Wien, Höchstädtplatz 6, 1200 Wien, Austria.

CONTENTS:

1. LEEM analysis of UHV-exfoliated Bi_2Se_3(0001)

2. Determination of Bi_2Se_3 surface termination

3. Bright-field image of Fe-DCA on Bi_2Se_3(0001)

4. STM for submonolayer Fe-DCA coverages

5. STM images of Fe-DCA with different tip conditions

6. Mixed honeycomb-kagomé Fe_2DCA_3 on Au(111)

7. Off-stoichiometric candidate structures for the phase B

1. LEEM analysis of UHV-exfoliated $Bi_2Se_3(0001)$

A freshly exfoliated Bi_2Se_3 crystal was analyzed by LEEM/LEED, XPS, and STM to assess surface quality. Detailed analysis by XPS and STM is given in ref. 1. Here, we focus on LEEM analysis. The exfoliated surface shows variations in real space between individual exfoliations, as detailed below. Despite the real-space variation, sharp spots in the diffraction pattern in Figure S1a reflect the three-fold symmetry of the quintuple layer and indicate a clean substrate. In real space (Figure S1b), the brighter areas are terraces (marked by green arrow) separated by step edges visualized by dark lines marked by yellow arrow; the dark circular features (blue arrow) are associated with bismuth clusters (see Section S2); their presence and number vary with each exfoliation; these clusters appear when Bi precipitates from the bulk interstitial positions to the surface.

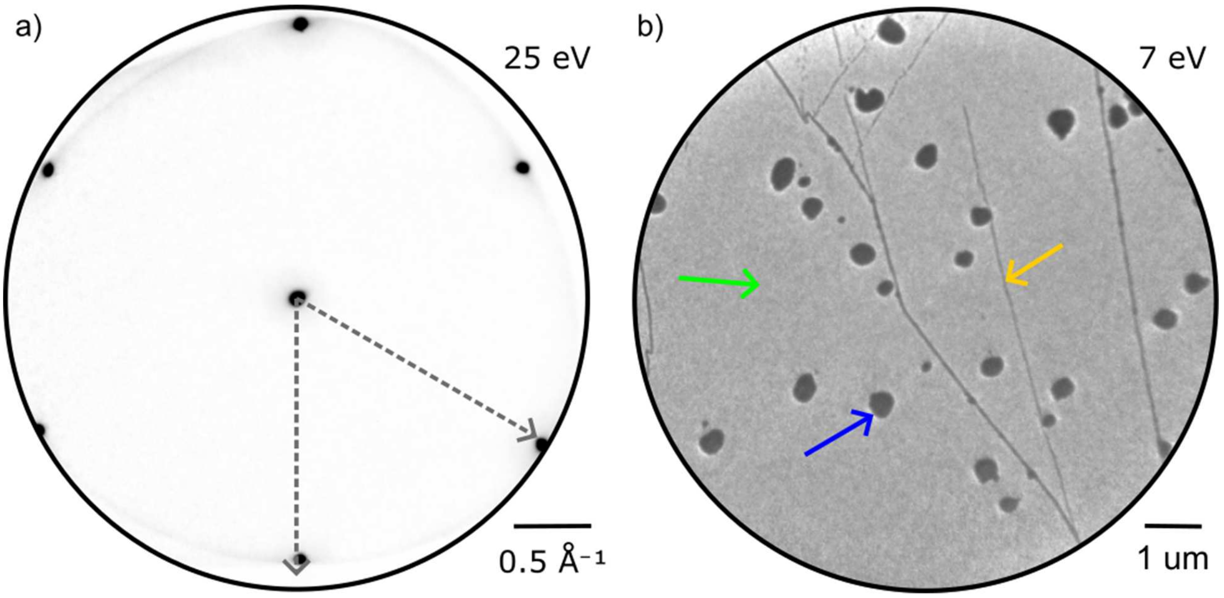

Figure S1: Freshly exfoliated $Bi_2Se_3(0001)$ crystal characterized in LEEM/LEED. (a) The LEED pattern shows clear hexagonal symmetry. (b) Bright-field LEEM image providing a real-space view of the exfoliated surface, with highlighted features: green arrow indicates large terraces, yellow points to step edges, and blue highlights circular dark features present after exfoliation, assigned to Bi clusters precipitated from the bulk.

2. Determination of Bi_2Se_3 surface termination

An exfoliated surface is supposed to be Se-terminated and therefore interact weakly with adsorbates.[2] However, there has been some controversy around this topic, as a few articles reported Bi-rich termination or Bi-bilayer termination.[3–7] The appearance of this termination depends mainly on the crystalline quality (i.e., the growth process) and, to a lesser extent, on the cleaning process and sample storage conditions. To demonstrate that our substrate is indeed Se-terminated and to determine the origin of clusters observed on the exfoliated surface (Section S1), we have performed a series of experiments involving the deposition of Bi atoms onto a freshly exfoliated Bi_2Se_3 sample.

Upon the deposition of Bi, we observe growth of dark clusters in the LEEM bright field, as highlighted with a red arrow in Figure S2a and b. These clusters have a very similar appearance to those on the freshly exfoliated surface (blue arrow), suggesting a bismuth origin for the latter. We have compared LEEM-IV curves for these two clusters (Figure S2c). Because the LEEM-IV curve is highly sensitive to structure and composition, it can serve as a material and structural fingerprint.[8] Although the curves do not match in amplitude, the curves match in position and relative height of all peaks and other features. This indicates that the clusters observed after exfoliation are composed of Bi, consistent with the initial hypothesis.

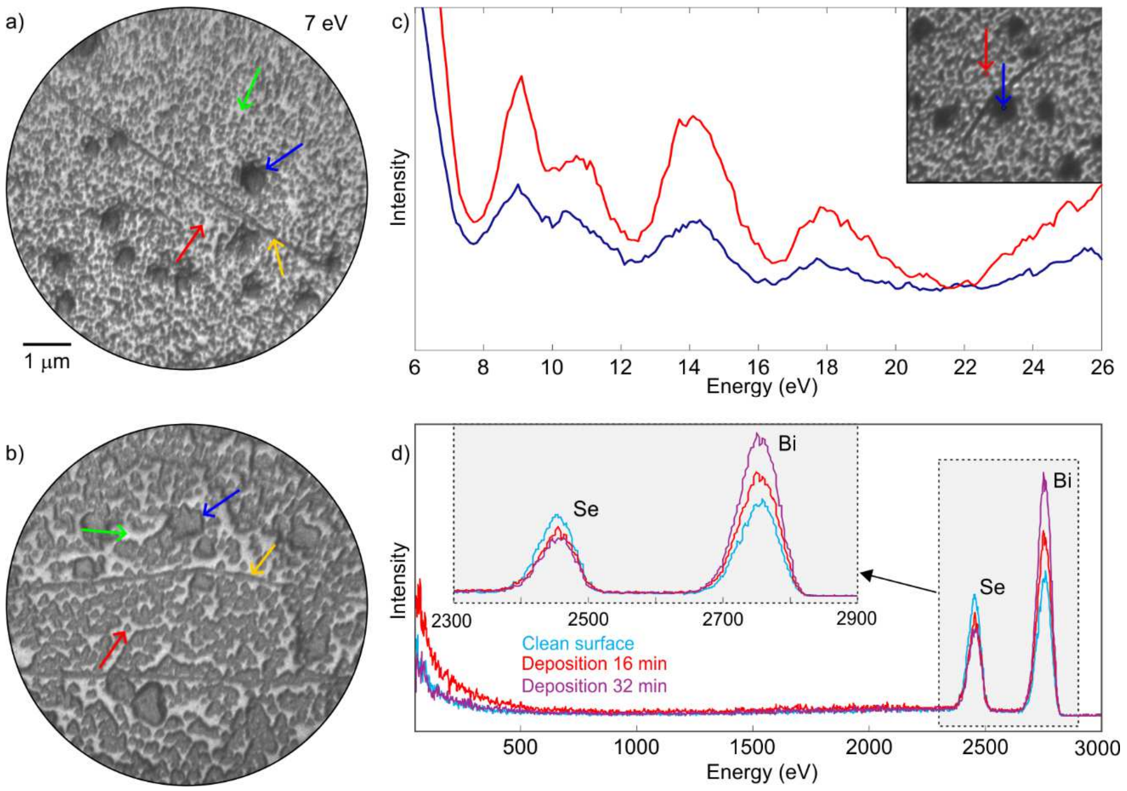

Figure S2: (a, b) Bright-field LEEM images taken at 7 eV on $Bi_2Se_3(0001)$ after deposition of Bi for 16 min (a) and 32 min (b). The green arrow highlights terraces, the yellow points to step edges, the blue highlights circular dark features present before exfoliation, and the red indicates deposited Bi clusters. (c) LEEM-IV curves acquired on clusters present before Bi deposition (blue) and those that appeared after deposition (red) as marked in the inset; the curves are plotted for a single pixel of the image after alignment of the image series with sub-pixel precision. (d) LEIS spectra measured with He ions on a freshly exfoliated surface (Figure S1b), after 16 min (Figure S2a), and 32 min of Bi deposition (Figure S2b).

We have employed low-energy ion scattering spectroscopy (LEIS) to determine the composition of the topmost surface layer of the in-situ exfoliated Bi_2Se_3 samples. LEIS spectra were measured on a freshly exfoliated Bi_2Se_3 surface (corresponding to Figure S1), and after 16 and 32 min of Bi deposition, corresponding to Figures S2a and b, respectively. Spectra were acquired at four distinct sample locations at each stage, showing consistent results. Even on a freshly cleaved surface, we

observe a significant Se peak, suggesting that Bi bilayer termination is very unlikely. With increasing Bi coverage, the Bi peak increases and the Se peak decreases in the LEIS spectra shown in Figure S2d. However, despite the high coverage of Bi clusters, there is still a quite high signal of the Se peak, likely associated with uncovered areas on the sample as marked by the green arrow in Figure S2a and b.

In the LEEM bright-field images, the lateral growth of the Bi cluster slows significantly during deposition. Accordingly, the increase in Bi LEIS intensity and the decrease in Se intensity are not proportional, as summarized in Table S1. Compared to the clean sample, the Bi signal increases by 33% after 16 min and 74% after 32 min of Bi deposition, whereas the Se peak decreases by 20% and 26%, respectively. Hence, the increase in Bi between two subsequent depositions is not accompanied by a comparable decrease in Se intensity. This points to the formation and growth of 3D clusters.

In summary, LEIS measurement suggests that Bi atoms form clusters on the Bi_2Se_3 surface, instead of a homogeneous bilayer, leaving uncovered Se-terminated areas. This points towards Se-termination of the freshly exfoliated surface.

Table S1: Comparison of Se and Bi peak intensities in LEIS spectra and their ratios for each step. Intensity values are relative to the freshly exfoliated surface. The data are treated only qualitatively by comparison with the clean substrate; a quantitative analysis was not performed due to the lack of suitable reference data for the individual elements.

Deposition time	Se peak area (%)	Bi peak area (%)	Se/Bi ratio
0 min	100	100	0.77
16 min	80	133	0.53
32 min	74	174	0.33

3. Bright-field image of Fe-DCA on Bi_2Se_3(0001)

There is a visible change in contrast in the LEEM bright-field image in Figure S3 after deposition compared to the clean surface. The image shows an overall reduced contrast, suggesting the presence of an overlayer, even though the individual islands cannot be resolved.

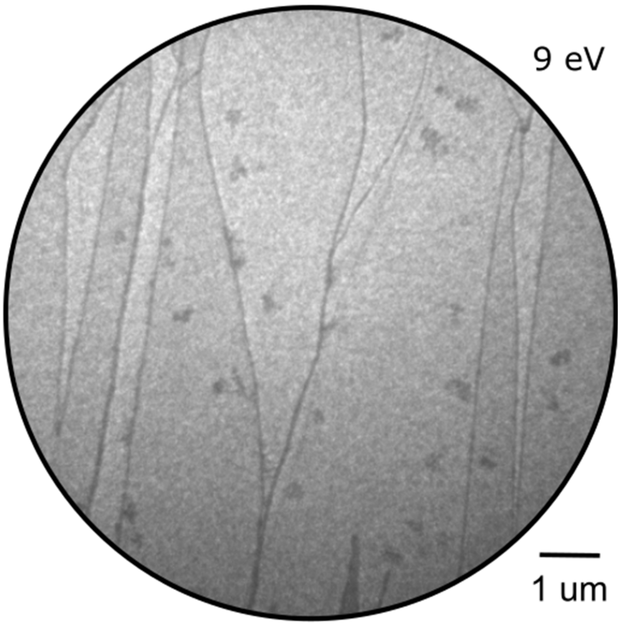

Figure S3: Bright-field LEEM image providing a real-space view of the Bi_2Se_3(0001) after deposition of Fe-DCA.

4. STM for submonolayer Fe-DCA coverages

While STM measurements at room temperature were very unstable for submonolayer Fe-DCA coverages, they still suggest the presence of a periodic arrangement, as shown in Figure S4a. After cooling to –100 °C with liquid nitrogen, molecular resolution was achieved, as shown in Figure S4b, but significant drift prevented precise length measurements.

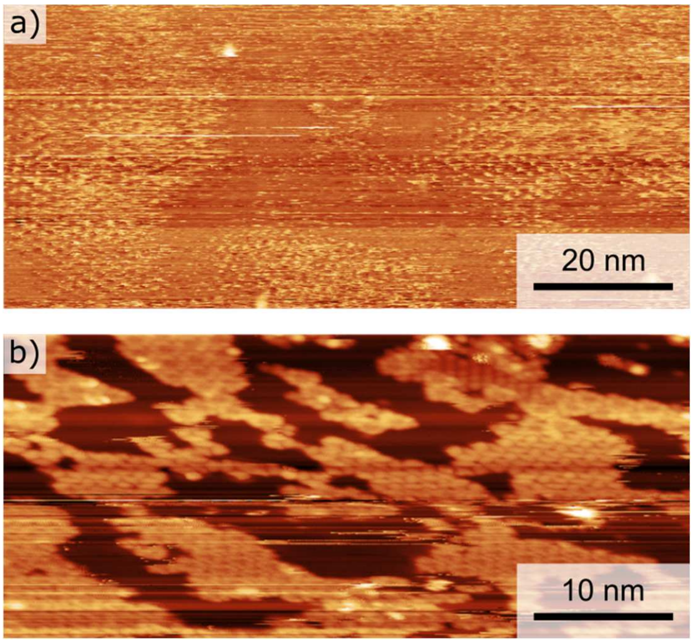

Figure S4: STM images measured for submonolayer coverage on a sample featuring phase B on the $Bi_2Se_3(0001)$ surface. (a) Room temperature image; atomic resolution was not reached. (b) Low temperature (−100 °C) STM image. The structure is composed of clover-leaf motifs arranged in a hexagonal lattice, forming islands up to 10 nm in size. Scanning parameters: (a) 1.0 V, 30 pA and (b) 3 V, 90 pA.

5. STM images of Fe-DCA with different tip conditions

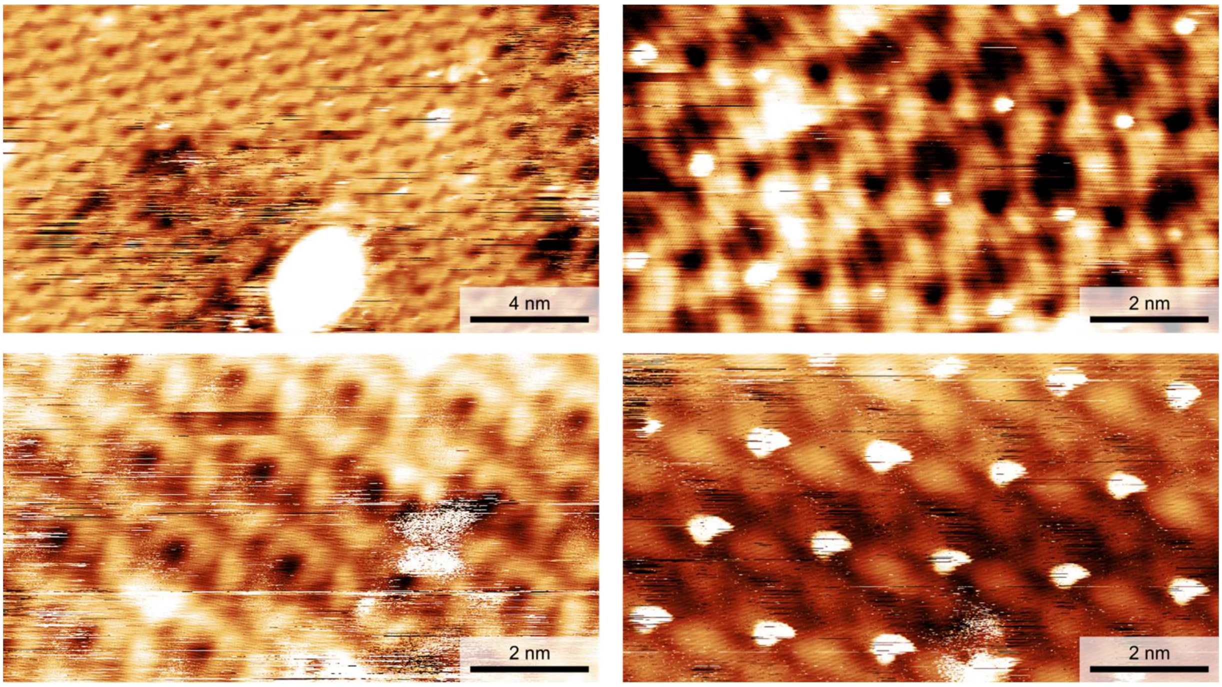

Figure S5: STM images showing different appearances of the clover-leaf motif with different tip conditions.

6. Mixed honeycomb-kagomé Fe_2DCA_3 on Au(111)

Fe_2DCA_3 with MHK structure prepared on Au(111) is shown in Figure S6. At some point, the tip conditions changed, revealing a different view of the MHK lattice that resembles the clover-leaf motif. However, compared to Fe-DCA on Bi_2Se_3 in Figure S7, the angle between the individual clover motifs (marked by triangles) is different for the MHK structure on Au(111) and phase B on Bi_2Se_3. This suggests that phase B likely does not have an MHK structure.

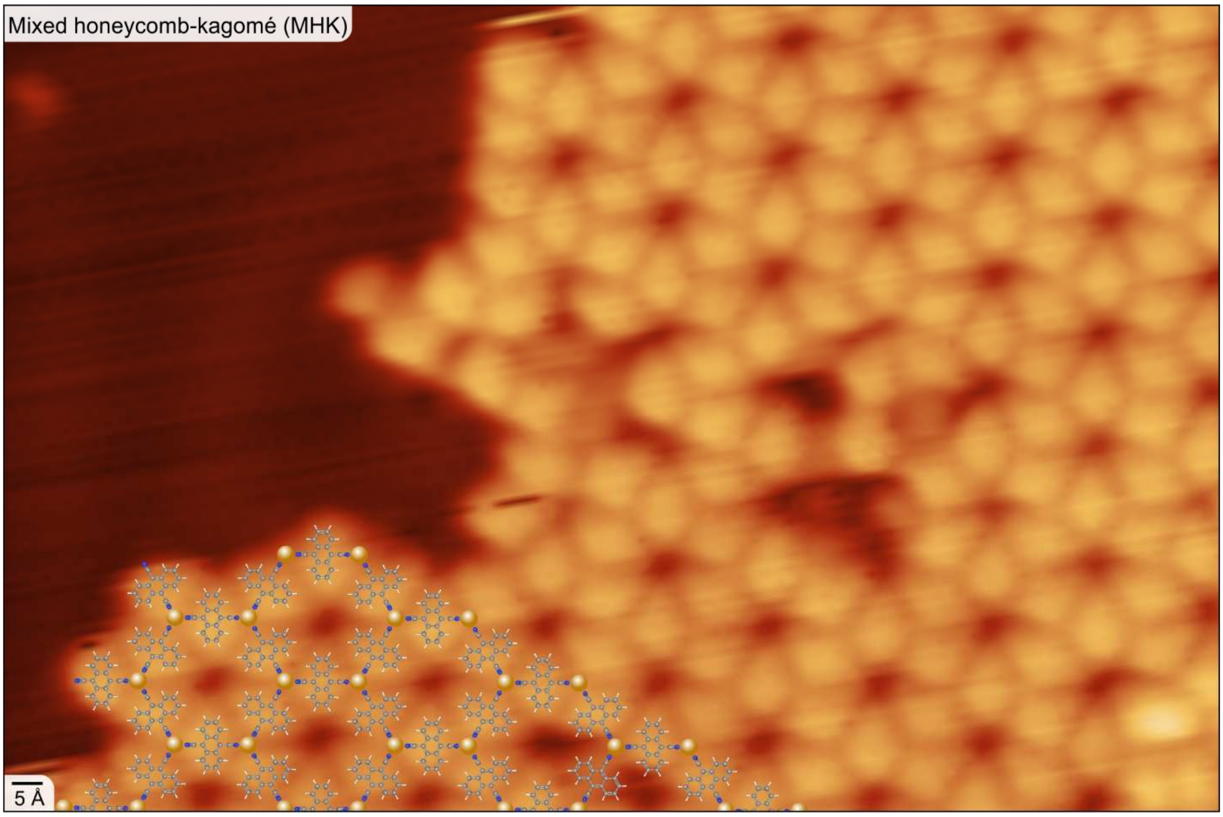

Figure S6: High-resolution STM images of Fe-DCA structure on Au(111) surface showing MHK structure of Fe_2DCA_3.

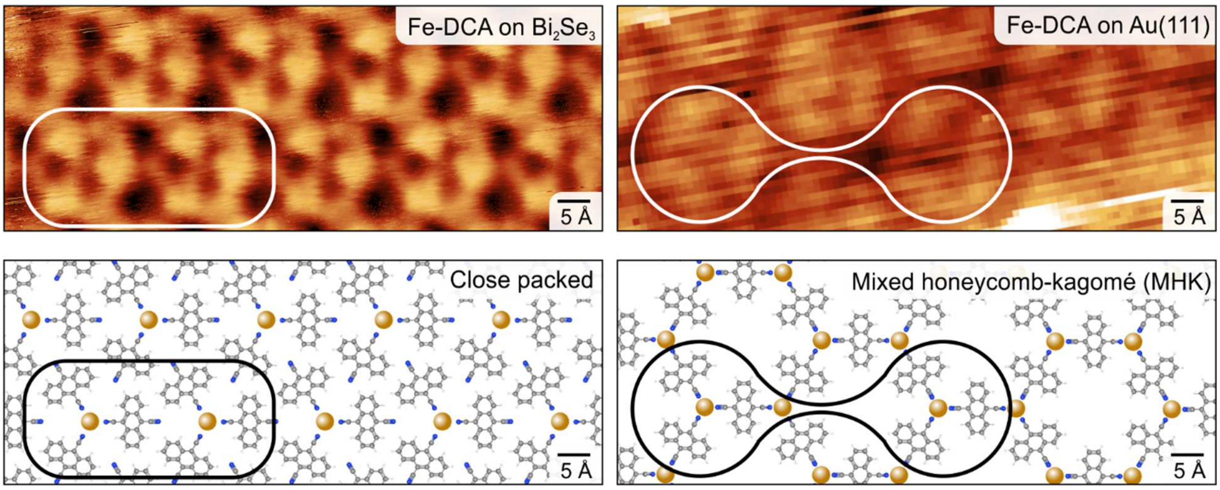

Figure S7: Comparison of STM images of Fe-DCA on $Bi_2Se_3(0001)$ and Au(111). At certain tip conditions, the MHK on an Au(111) is visualized similarly to clover-like objects. These have distinct mutual orientations as compared to clover-like structures observed on Bi_2Se_3.

7. Off-stoichiometric candidate structures for the phase B

This section provides additional information about the calculated off-stoichiometric structures that could potentially explain the misalignment between theory and experiment for phase B. Specifically, we considered structures similar to the clover phase that are stabilized by the following additional features: (i) a selenium adatom, (ii) a bismuth adatom, and (iii) small Fe_3Se_x clusters. The most stable configuration with an additional selenium adatom is shown in Figure S8A, where the Se adatom binds to the free carbonitrile group of FeDCA. The calculated binding energy is, however, 1.2 eV weaker than in bulk selenium, and the clover structure undergoes significant bending that would likely be observable in STM. We therefore conclude that such a stabilization mechanism is unlikely.

Regarding Bi adatoms, we calculated the stability of the clover phase with the free carbonitrile group terminated by an additional Bi atom (Figure S8B) and compared it with the MHK structure in which the second Fe atom is replaced by Bi (Figure S8C). Since the overall stoichiometry of both systems is identical, their stability can be directly compared using the total energies. In this case, the MHK-like structure is 600 meV more stable. Hence, we conclude that Bi adatoms cannot explain the structure of phase B either.

Lastly, motivated by a previous study indicating a strong interaction between Fe and Se that can lead to the formation of an FeSe monolayer on top of the Bi_2Se_3 substrate,[9] we replaced the central Fe atom by an Fe_3Se_x cluster (x=3,4), structurally similar to those reported in Ref. 10. The resulting structure, shown in Figure S8D, has an optimal lattice parameter of 19.4 Å, i.e., very close to the experimental value of 19.0 Å. Unfortunately, the stability of such off-stoichiometric structures cannot be compared directly with the clover phase, since the chemical potentials of the additional species are experimentally unknown. Although the presence of such clusters is, out of

the considered models, the most promising explanation, it would need to be verified by additional experimental outputs.

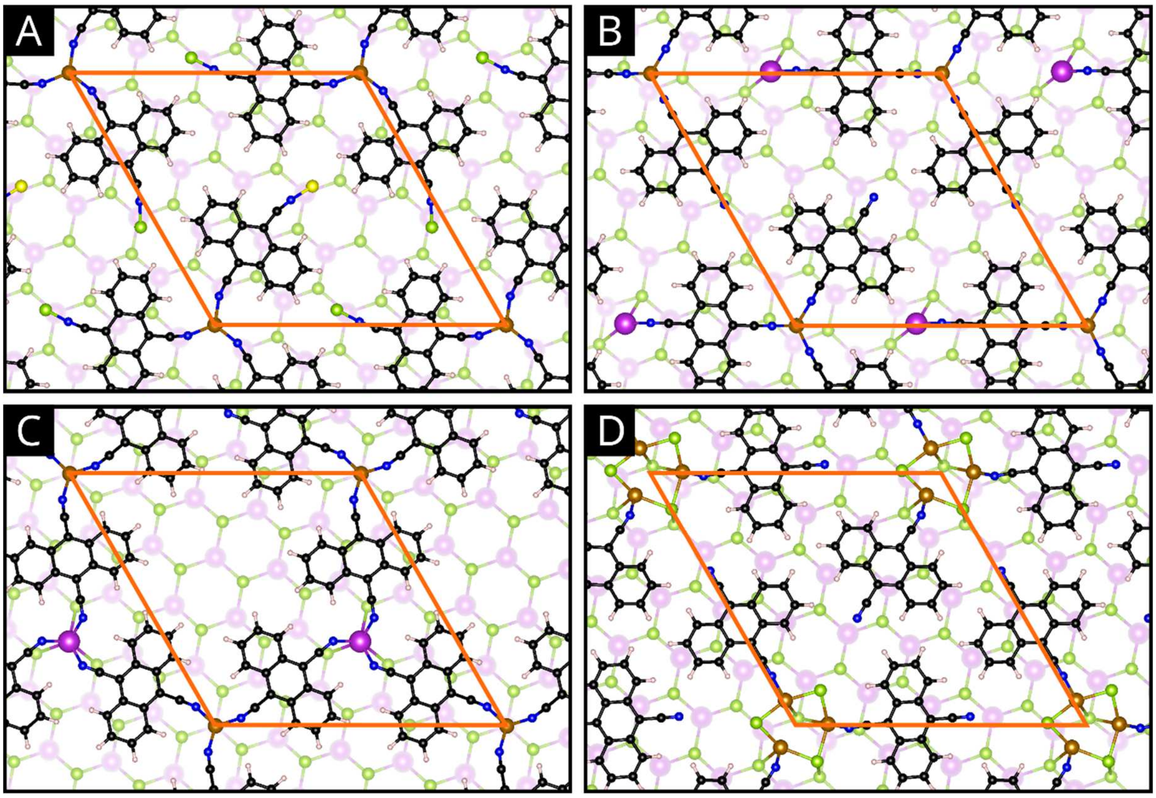

Figure S8: Calculated clover structures with different adatoms, using the unit cell of the phase B. Atoms are colored black (carbon), pink (hydrogen), beige (iron), violet (bismuth), and green (selenium). Panel (A) shows the Se-terminated FeDCA clover, with the additional Se atom highlighted in yellow. Two examples of Bi-rich configurations with the same stoichiometry are shown in panels (B) and (C), respectively: the Bi-terminated FeDCA clover and the MHK structure with one Fe atom replaced by Bi. Extension of the central Fe atom into an Fe_3Se_3 cluster is shown in panel (D).

References

(1) Čechal, J.; Stará, V.; Kurowská, A.; Blatnik, M.; Pravec, D.; Hrubá, D.; Kunc, J.; Drašar, Č.; Planer, J.; Procházka, P. Structural, Electronic, and Chemical Manifestations of Weak Molecule–Substrate Coupling on Bi_2Se_3 Compared with Ag, Au, and Graphene. *Appl. Surf. Sci. Adv.* **2026**, *32*, 100927. https://doi.org/10.1016/j.apsadv.2025.100927.

(2) Zhang, Y. N. Communication: Surface Stability and Topological Surface States of Cleaved Bi_2Se_3: First-Principles Studies. *J. Chem. Phys.* **2015**, *143* (15), 151101. https://doi.org/10.1063/1.4933298.

(3) Zhou, W.; Zhu, H.; Yarmoff, J. A. Termination of Single-Crystal Bi_2Se_3 Surfaces Prepared by Various Methods. *Phys. Rev. B* **2016**, *94* (19), 195408. https://doi.org/10.1103/PhysRevB.94.195408.

(4) Deng, Q.; Pan, J.; Yin, X.; Wang, X.; Zhao, L.; Kang, S. G.; Jimenez-Cruz, C. A.; Zhou, R.; Li, J. Toward High Permeability, Selectivity and Controllability of Water Desalination with FePc Nanopores. *Phys. Chem. Chem. Phys.* **2016**, *18*, 8140–8147. https://doi.org/10.1039/c6cp00322b.

(5) Hewitt, A. S.; Wang, J.; Boltersdorf, J.; Maggard, P. A.; Dougherty, D. B. Coexisting Bi and Se Surface Terminations of Cleaved Bi_2Se_3 Single Crystals. *J. Vac. Sci. Technol. B, Nanotechnol. Microelectron. Mater. Process. Meas. Phenom.* **2014**, *32* (4), 04E103. https://doi.org/10.1116/1.4873689.

(6) Edmonds, M. T.; Hellerstedt, J. T.; Tadich, A.; Schenk, A.; O'Donnell, K. M.; Tosado, J.; Butch, N. P.; Syers, P.; Paglione, J.; Fuhrer, M. S. Stability and Surface Reconstruction of Topological Insulator Bi_2Se_3 on Exposure to Atmosphere. *J. Phys. Chem. C* **2014**, *118* (35), 20413–20419. https://doi.org/10.1021/jp506089b.

(7) Coelho, P. M.; Ribeiro, G. A. S.; Malachias, A.; Pimentel, V. L.; Silva, W. S.; Reis, D. D.; Mazzoni, M. S. C.; Magalhães-Paniago, R. Temperature-Induced Coexistence of a Conducting Bilayer and the Bulk-Terminated Surface of the Topological Insulator Bi_2Te_3. *Nano Lett.* **2013**, *13* (9), 4517–4521. https://doi.org/10.1021/nl402450b.

(8) Čechal, J.; Procházka, P. Low-Energy Electron Microscopy as a Tool for Analysis of Self-Assembled Molecular Layers on Surfaces. *J. Phys. Condens. Matter* **2025**, *37*, 293003. https://doi.org/10.1088/1361-648X/ade946.

(9) Fikáček, J.; Stetsovych, V.; Vondráček, M.; Procházka, P.; Průša, S.; Kormoš, L.; Čechal,

J.; Caha, O.; Skála, T.; Vlaic, P.; Carva, K.; Springholz, G.; Honolka, J. Step-Edge Assisted Large Scale FeSe Monolayer Growth on Epitaxial Bi_2Se_3 Thin Films. **2019**.

(10) Dahl, L. F.; Sutton, P. W. Structure of Se 2 Fe 3 (CO) 9 and Evidence for a New Type of Seven-Coordinated Metal. *Inorg. Chem.* **1963**, *2* (5), 1067–1069. https://doi.org/10.1021/ic50009a047.
